# Fault Tolerant DHT-Based Routing in MANET

**DOI:** 10.3390/s22114280

**Published:** 2022-06-03

**Authors:** Saleem Zahid, Kifayat Ullah, Abdul Waheed, Sadia Basar, Mahdi Zareei, Rajesh Roshan Biswal

**Affiliations:** 1 Institute of Computer Science & Information Technology, FMCS, The University of Agriculture, Peshawar 25130, Pakistan; szahid77@gmail.com; 2 Department of Computer Science, CECOS University, Peshawar 25000, Pakistan; kfytullah@gmail.com; 3 Department of Computer Science, Northern University, Nowshera 24100, Pakistan; 4 School of Electrical and Computer Engineering, Seoul National University, Seoul 08826, Korea; 5 Department of Information Technology, Hazara University Mansehra, Mansehra 21120, Pakistan; sadiaa.khancs@gmail.com; 6 School of Engineering and Sciences, Tecnologico de Monterrey, Zapopan 45201, Mexico; m.zareei@tec.mx

**Keywords:** Distributed Hash Table (DHT), fault tolerance, Mobile Adhoc Networks (MANET), routing

## Abstract

In Distributed Hash Table (DHT)-based Mobile Ad Hoc Networks (MANETs), a logical structured network (i.e., follows a tree, ring, chord, 3D, etc., structure) is built over the ad hoc physical topology in a distributed manner. The logical structures guide routing processes and eliminate flooding at the control and the data plans, thus making the system scalable. However, limited radio range, mobility, and lack of infrastructure introduce frequent and unpredictable changes to network topology, i.e., connectivity/dis-connectivity, node/link failure, network partition, and frequent merging. Moreover, every single change in the physical topology has an associated impact on the logical structured network and results in unevenly distributed and disrupted logical structures. This completely halts communication in the logical network, even physically connected nodes would not remain reachable due to disrupted logical structure, and unavailability of index information maintained at anchor nodes (ANs) in DHT networks. Therefore, distributed solutions are needed to tolerate faults in the logical network and provide end-to-end connectivity in such an adversarial environment. This paper defines the scope of the problem in the context of DHT networks and contributes a Fault-Tolerant DHT-based routing protocol (FTDN). FTDN, using a cross-layer design approach, investigates network dynamics in the physical network and adaptively makes arrangements to tolerate faults in the logically structured DHT network. In particular, FTDN ensures network availability (i.e., maintains connected and evenly distributed logical structures and ensures access to index information) in the face of failures and significantly improves performance. Analysis and simulation results show the effectiveness of the proposed solutions.

## 1. Introduction

In general, MANET routing protocols use IP addresses for nodes identification as well as for routing and consider node identity equal to routing address, i.e., static addressing. These identifiers are independent of the relative location of nodes in the network and do not provide any information to guide the routing process. Therefore, these protocols rely on flooding or network-wide routing information dissemination and degrade performance as a network scales. On the contrary, the identity and location of nodes should be considered separately because nodes are mobile, and the network topology continuously changes. Therefore, the nodes should have a transient routing address that reflects their relative position concerning their neighbor nodes. From this emerges the concept of dynamic or transient addressing, where a node changes its address according to its location. In this manner, the transient addresses build a logical, structured network that guides the routing process. This eliminates flooding in routing and makes the system scalable. DHT provides a scalable way to decouple node location from its identity and facilitate general mapping between them. In the last few years, several DHT-based routing protocols for MANETs have been proposed that eliminate flooding in the route discovery, thus making the network more scalable [1,2,3,4,5,6,7,8,9,10,11,12,13,14,15,16,17,18]. In DHT-based routing protocols, a node is assigned a logical identifier (LID) in addition to its permanent identifier (i.e., MAC/IP address also known as user id, i.e., UID) based on the LIDs of its physical neighbors. The LID is drawn from a predefined logical identifier space (LS) (i.e., the LS forms a logical, structured network following a tree, ring, chord, etc., structure). A node must maintain a disjoint portion of the whole LS, referred to as Logical Space Portion (LSP) (i.e., the LSP acts as the LID for a node). Moreover, each node keeps track of its logical neighbor nodes with LIDs close to its LID by following a ring, chord, or multidimensional structure. Thus, a logical, structured network is built over the ad hoc physical topology. Routing is performed in the logical, structured network using the transient addresses (i.e., LIDs). The logical structure guides routing processes and eliminates flooding at the control plane and the data plane. Furthermore, a connected and evenly distributed LS is always needed for successful routing.

**Figure 1:**
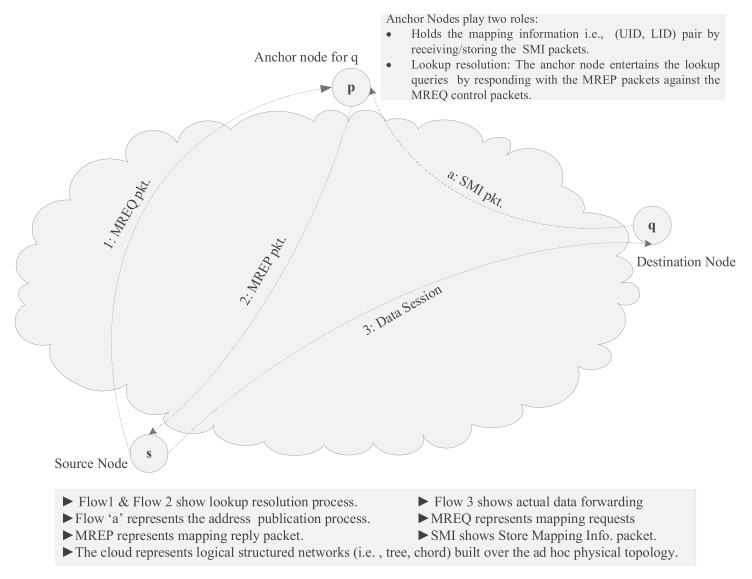
Address publication, lookup, and routing in DHT networks.

DHT networks can be defined with the help of three entities: (i) Logical Network construction, (ii) Address Publication phase, also called Anchor Request, and (iii) Lookup phase, also known as Address Resolution, and actual data forwarding.

i. Logical network construction: In network bootstrapping, nodes compute LIDs and form a logical, structured network (i.e., LS) over the ad hoc topology of MANET. For this purpose, a periodic hello message containing necessary information (depends on protocol specification) is exchanged among the neighboring nodes.
ii. Address Publication/ Anchor Request: After computing LIDs, nodes store their index information, also called mapping information (i.e., (LID, UID) pair), by sending store mapping information (SMI) packets, at certain nodes in the network referred to as an anchor node (AN). A node determines its AN by applying a hash function over its UID and generates a hashed value, h(v). The hashed value h(v) is drawn from the same LS used to assign LIDs to nodes. For example, a node p would act as the AN for node q if either h(v) falls in the LSP of node p or node p’s LID is closest to h(v), depending on the protocol specification. The SMI packet is unicasted in the logical network to node q, and node q stores index information for node p. (i.e., node q starts acting as a designated AN for node p). The address publication process is shown in Figure 1, dotted line in the figure represents the address publication process.
iii. Lookup phase and actual data forwarding: To send a data packet, a source node s retrieves the destination node q’s LID from q’s AN, i.e., node p, (Flow 1 and Flow 2 in Figure 1 shows the lookup process). For this purpose, node s applies a hash function over q’s UID that gives the LID of node q’s AN, i.e., node p. Based on the computed LID, node s sends a mapping request message (MREQ) towards node p forwards to get the index information of node q. Upon receiving the index information of node q on the mapping reply message (MREP), node s then sends the data packet towards node q based on the node q LID; Flow 3 in Figure 1 shows data packet routing.

Greedy forwarding is used in the logical, structured network, i.e., the neighbor with the closest LID to the intended destination node is selected as the next hop. However, successful resolution of lookup queries, with minimum possible delay, is imperative for optimal network performance. The terms used in the paper are defined in Table 1.

In DHT networks, logical network construction, address publication, and lookup processes are the main factors and define overall network performance. Therefore, sophisticated techniques are needed to keep the system in function, especially in the adversarial environment offered by MANETs. MANETs are entirely self-organized, mobile, distributed, and infrastructure-less networks. Nodes in the network with limited transmission ranges use wireless broadcasting mechanisms in a multi-hop fashion to achieve end-to-end connectivity. Limited radio range, mobility, faulty nodes, and lack of infrastructure introduce frequent and unpredictable changes to network topology, i.e., connectivity/dis-connectivity (can cause occasional nodes failures, network partition, frequent merging, etc.). Therefore, an end-to-end path that is believed optimal at a given time might not be available just after a moment. The case is more severe in DHT networks, where a logical, structured network is built over the ad hoc physical topology, and routing needs an evenly distributed and connected logical network. Similarly, access to the index information stored at ANs (i.e., lookup queries resolution) is imperative for successful routing. Another correlated problem is the longer lookup delay in DHT routing, where network dynamics significantly exacerbate the delay. Furthermore, frequent merging is common in MANETs, but DHT needs extra effort to merge logical structures/LSs (i.e., LIDs reconfigurations, etc.) in a distributed manner. The impact of network dynamics on DHT networks with a detailed explanation is discussed in Section 2.

It is worth mentioning that the problem of fault tolerance, in the context of DHT networks, has not yet been fully explored. There exist no explicit and direct solutions to the problem. To the best of author’s knowledge, existing DHT-based routing protocols in MANETs [1,2,3,4,5,6,7,8,9,10,11,12,13,14,15,16,17,18] mainly consider routing and resilience of the address spaces used. In other words, existing protocols do not consider network dynamics and associated issues that limit the scope of these protocols in the adversarial environment of MANET. However, some closely related work [13,14] consider the problem partially and solve it independently in the logical network without using physical network information. The adopted mechanisms in [13,14] introduce huge lookup delays with extra overhead. For further detail please see Section 3.

The paper contributions are twofold, i.e., in the problem domain and the solution domain. In particular, the main contributions are summarized as under:

- This paper explores the classical problem of fault tolerance in DHT-based MANET and defines its scope(Section 2). In particular, issues such as disrupted logical network structures, loss of index information due to AN failure or network partition, detecting and differentiating occasional node failure (i.e., node failure without causing network partitioning, where alternate paths avoiding the failing node are needed to be found) and partition detection, merging of logical structures and LID reconfiguration are addressed (for details, please see Section 2). Additionally, limitations of the existing protocols are highlighted in Section 3.
- In the solution domain, a suite of distributed mechanisms is provided for the identified problems under the scalability constraints. Specifically, we consider k-hop topological information and the philosophies of detection, recovery, avoidance, and redundancy to cope with the problems. Moreover, pre-failure measures (finding critical regions in the network, dynamic replication, and partition detection) and post-failure measures (finding alternate paths avoiding the failure, merging and merging detection, and LIDs reconfigurations) are considered in a distributed manner. (Section 4 presents detailed description).

The rest of the paper is structured as follow: the scope of the problem is defined in Section 2, and related work is provided in Section 3. Section 4 presents a suite of distributed solutions. Simulation and results analysis are given in Section 5. Finally, Section 6 concludes the paper.

## 2. The Problem and Its Scope

Generally in MANETs, existing literature [19,20,21,22,23] on the fault tolerance (an overview is provided in Section 3) targets the node(s) failure problem and assumes that a subset of nodes in the network fail. The routing process avoids such faulty nodes and makes packet delivery possible. The existing solution domain is based on the design philosophies of detection, avoidance, and redundancy. The employed mechanisms detect faulty nodes and avoid such nodes in the routing process. Similarly, in redundancy-based mechanisms, multiple routes are maintained between a source and destination pair, where a stable path, i.e., having no faulty nodes, is selected for routing. However, the case is different in DHT networks where a logical, structured network is built and maintained over the underline ad hoc physical topology. The relationship between the physical network and the logical network is tight. In other words, a change in the physical network has an associated impact on the logical, structured network. Similarly, node failure has an associated impact on the logical, structured network. For example, a single fault in the physical topology is directly associated with the loss of index information (if AN fails, AN holds index information), logical network partition (i.e., LS loss and disrupted LSs in the created instances), and indirectly with merging. These problems halt communication entirely and need to be addressed for optimal network performance. We further explore the problem in DHT networks. Our findings are summarized as below:

- Existing solutions for the fault tolerance problem are purely designed for flooding-based infrastructure-less MANET and are not applicable in DHT-based MANET.
- We found that existing solutions [19,20,21,22,23], including multi-path routing, blindly assume a fault ratio and do not investigate the failure (as not needed). However, in a DHT network, a node failure causes two different events: (i) occasional node failure without creating a partition, alternate paths avoid the failure, and routing processes should avoid the failure in forwarding decisions. Moreover, in (ii) node failure causing network partition, there exist no alternate paths at all. Similarly, both events have a different associated impact on the DHT network. For example, in case of event (i) if the failing node is AN, then the corresponding mapping information remains unreachable. Similarly, in case (ii), node failure would cause loss of mapping information and leave unevenly distributed and disrupted LSs (logical networks) in the disjoint instances. This halts communication in the disjoint partitions. Therefore, mechanisms are needed to detect and differentiate such events in DHT networks. Algorithms 3 and 4 address the problems using k-hop topological information, respectively.
- Lookups and AN failure: In the lookup phase, source nodes retrieve routing information stored on ANs to initiate a data session with destination nodes. The lookup request initiated by the source node is routed towards intended ANs in the network. The designated AN resolves the query by forwarding the required routing information. Therefore, successful lookup resolution, with minimum possible delay, is imperative in DHT-based routing. Moreover, DHT inherently suffers from longer lookup delays in the lookup phase. However, network dynamics prohibit lookup resolution and introduce massive delays. For example, in the case of AN failure (due to occasional node failure or network partition), the mapping information is lost, and corresponding lookup queries would not be entertained. The requesting party will keep trying to resolve the query, even the AN is no longer available. This causes the failure of lookup and introduces massive lookup delays and overhead. A similar situation arises, for example, when a source and destination pair remains in one partition, and the corresponding AN resides in a disjoint partition after the network gets partitioned. In short, the lookup phase defines the overall performance of DHT networks and needs mechanisms to ensure access to mapping info. In the presence of failure/partition. Algorithm 2 performs replication decisions across the critical regions (identified by Algorithm 1 in a dynamic and distributed manner and ensures access to mapping info in the adversarial environment.
- Merging: Similarly, we argue that merging is indirectly associated with node failure (i.e., failure causing network partition) and should be treated as an integral part of any fault-tolerant protocol in DHT networks. Secondly, merging is different in DHT (i.e., need to merge disjoint logical structure (LS) to achieve an evenly distributed and connected LS). For example, in the case of a network partition, there would exist multiple network instances. In a mobile and self-organized environment, frequent merging might take place. Simple merging (i.e., physical topology) of the disjoint instances would not restore communication in a DHT-based network. Instead, it will result in a physically connected but logically disconnected network [5] (i.e., need to merge the logical structures for an evenly distributed LS). Therefore, additional measures such as LIDs reconfiguration, root election, etc., are needed to obtain uniformly distributed and connected LS. Moreover, merging detection in the physical network is needed to initiate merging in the logical network. It is pertinent to mention that existing merging solutions [5] assume uniformly distributed LSs in the disjoint networks for smooth merging. However, partitioning leaves unevenly distributed and disrupted instances of the logical network (LS). In fact, due to network partition, a portion of the logical network (LS) is lost. Therefore, for smooth operations in the disjoint instances and smooth merging, uniformly distributed LSs are needed. To solve the problem, the lost LS needs to be recovered and reused in the created instances after a network partition. LS recovery and reusing purely depend on protocol specification. For example, in tree shape LS, the lost LS is recovered and reused by re-electing new representative nodes, LIDs reconfigurations, etc., in the disjoint instances. However, LS recovery/reusing needs timely detection of the partition event. Therefore, partition detection is another sub-problem in this particular domain. Algorithms 4 and 5 solve these problems in a distributed manner.

Therefore, DHT protocols should consider the above-highlighted issues to ensure optimal network performance in adversarial environments.

## 3. Related Work

Much research exists over the classical fault-tolerance problem in MANETs but is not applicable in DHT networks (for detail please see Section 2).

E2FT [19] addresses the problem using multipath source routing philosophy in MANET. The protocol maintains multiple paths between a source and destination pair. The protocol iteratively estimates and selects specific reliable paths among the available paths. For this purpose, a source node estimates the quality of a route by sending packets and measuring the delivery ratio (the destination node acknowledges the packets received). Specifically, the protocol uses mechanisms such as route estimation (a source node estimates the path quality, i.e., delivery ratio probability), and route selection (best paths are selected for routing with the help of two additional procedures known as confirmation and dropping). The confirmation and dropping procedures are used to refine the selection process progressively. However, the protocol incurs extra overhead to maintain a certain delivery ratio.

BFTR [20] performs routing in the presence of faulty nodes using a best-effort approach. BFTR’s key idea is to tolerate the fault to a certain extent and maintain maximal network performance in an adversarial environment. BFTR classifies existing routes between a source and destination into two classes, i.e., most feasible and non-feasible paths. For this purpose, the protocol maintains immediate records (i.e., delivery ratio and delay) of a path. The paths with the highest delivery ratio are marked as feasible and are selected for routing. Similarly, the paths having poor past statistics are declared as infeasible and are discarded. BFTR does not detect faulty nodes on a routing path, but evaluates a path’s routing feasibility based on its past end-to-end performance statistics.

An enhancement over E2FT [19] was proposed known as weak-estimation learning-based fault-tolerant routing (WEFTR) [21,22]. WEFTR exploits stochastic learning procedures and obtains better path estimation and selection. The protocol achieves a certain packet delivery ratio with relatively low overhead as compared to E2FT. WEFTR also argued that weak estimation procedures best suits mobile environments.

FTAR [23] proposed a bio-inspired solution to the problem of fault-tolerant routing in MANETS. FTAR exploits the ideas of foraging in natural ants [24]. A source routing mechanism is used to find routes between a source and destination pair. The proposed solution ranks the available paths using pheromone value/metric. In this manner, FTAR selects optimal routes (i.e., having minimum faulty nodes or high pheromone valued routes) among the existing routes.

However, existing DHT based routing protocols in MANETs [1,2,3,4,5,6,7,8,9,10,11,12,13,14,15] assume ideal network environments or mainly focus on addressing schemes and LS resilience in the perspective of routing. There exist no straightforward solutions, and the problem is not yet been explored in the DHT context. However, some closely related work partially targets and solves the problem (for certain specific scenarios); the subsequent paragraphs provide an overview of the closely related work.

VCP [13] exploits chord-shaped structures for DHT-based routing in MANETs. In network bootstrapping, nodes compute LIDs, in addition to UIDs, using a pre-defined LS that ranges [S = 0, E = 1]. In this manner, nodes build a logical chord-shaped structured network over MANET’s ad hoc physical topology. The LIDs computed based on adjacent physical neighbors’ LIDs show relative positions of nodes in the logical chord. Each node proactively maintains logical neighbors (predecessor/successor) and physical neighbors recorded in the routing table. Forwarding decisions consider physical and logical neighbors, where routing processes assume a uniformly distributed (i.e., dead-ends free) chord. Furthermore, in case of AN failure, VCP resolves lookup requests using redundancy. For this purpose, nodes forward and store mapping information on ANs in the address publication phase. Further, the designated ANs replicate and store copies at directly connected neighbors (i.e., 1-hop physical neighbors or logical neighbors predecessor/successor). VCP periodically updates the mapping information stored at actual ANs and the neighbors (i.e., replicas). So that, in case of an actual AN failure, lookup requests are routed to the vicinity of the failing node, and a ring search is employed to locate the replicas. The ring search is restricted to search replicas among 1-hop physical neighbors, i.e., using broadcast. There are several serious concerns regarding the fault tolerance capability of VCP. The replicas deployment, maintenance, and locating replicas introduce massive delays, overhead, and failures. Since VCP replicates and maintains (i.e., periodically updates) mapping information blindly and in a static manner (i.e., network dynamics are not considered in the replication decisions). This introduces extra overhead and delay. For example, VCP suffers from the mismatch problem [12,14]. Therefore, neighbors in the logical network might be distant nodes in the physical network and vice versa. So that replicating mapping information on predecessors/successors needs several transmissions in the physical network. This makes the replication process more expansive, mainly when VCP updates the replicas periodically. This also limits the applicability of the restricted ring search in the lookup process. The employed ring search, where the search space is restricted to 1-hop physical neighbors, misleads the lookup requests and introduces huge delay and overhead with false attempts to solve the lookup queries. Similarly, if the designated AN is critical and fails, then logical and physical neighbors would remain unreachable, or the network would get partitioned into disjoint parts. In these cases, the lookup resolution process would fail. Therefore, the replication decisions should be adaptive using knowledge about network dynamics. Furthermore, VCP does not consider network partition, merging, and its impact on network availability. An entirely fault-tolerant system should consider these practical considerations, especially in MANETs.

DART [14], and M-DART [15] embed the ad hoc physical topology of MANETs into a logical tree shape structured network. In network bootstrapping, joining nodes compute LIDs and form a logical tree-structured network (i.e., address tree). After computing LIDs, nodes store mapping information on specific ANs. A source node retrieves mapping information from the designated AN before starting the communication session with the destination node to route data packets. M-DART [15] is an enhanced version of DART. M-DART’s sole purpose was to explore and pro-actively maintain multiple paths in the address tree (i.e., logical network) between a source and destination pair. M-DART pro-actively and blindly (i.e., do not consider physical network connectivity) maintains multiple paths between a source and destination pair. Therefore, in case of a route failure, alternate paths, if available, can be exploited. This is an inherent feature of multi-path routing, i.e., redundancy. M-DART completely fails in situations where a single path exists. In other words, MDART does not consider network partition. Similarly, the impact of failures (i.e., AN failure, lookup failures, and address tree partitioning/merging) on DHT routing is not addressed. Moreover, M-DART does not claim fault tolerance capability and does not fit in the problem scope (please see Section 2). However, DART solves the problem partially by targeting the ANs failure problem. DART keeps updating mapping information on the ANs so that the information remains available after every update. The idea is to keep updating or selecting new ANs blindly and repeatedly. By doing so, the information will be available after every refresh period despite the failure. Similarly, if lookup queries arrive earlier than the updates (i.e., before selecting a new AN), the queries are buffered by the neighboring nodes and are forwarded towards the newly designated AN as soon as the updates arrive. This scheme has several problems; for example, it introduces an enormous delay (i.e., buffer delay, forwarding delay, and routing table updating time by the neighboring nodes) and overhead in the lookup resolution process. Further, the delay and overhead get worst as mobility and network size increase. Similarly, no buffer management is provided. However, mobility (high failure ratio) and network size would cause a buffer overflow, and lookup queries would get lost. Furthermore, DART mentioned the problems of partition and merging implicitly, using certain assumptions, without implementing algorithmic detail. These issues limit its scope in adversarial environments.

Another closely related work is [5], In [5], the merging problem is investigated in the context of mismatch [3,6] problem, and a leader election-based merging approach is utilized for merging DHT networks. However, the work assumes the existence of evenly distributed disjoint instances of the network, but this assumption does not hold in practice (for details, please see Section 2). Moreover, we use rank-based merging as a post-failure technique to recover from the failures and heal the logical, structured network. Additionally, FTDN ensures the existence of evenly distributed disjoint instances before initiating the merging process.

## 4. Fault Tolerant DHT Network (FTDN)

FTDN employs localized distributed algorithms and solves the problem of fault tolerance in a DHT-based MANET. FTDN, using a cross-layer design approach, investigates network dynamics in the physical network and adaptively makes arrangements to tolerate faults in the logical, structured DHT network. For this purpose, FTDN exploits k-hop topological information along with the philosophies of detection, recovery, avoidance, and redundancy. Specifically, FTDN considers pre-failure measures (i.e., measuring network dynamics, replication, and partition detection) and post-failure measures (i.e., finding alternate paths to avoid failure, merging detection, and reconfiguring). These measures ensure DHT network availability, successful lookup resolution and make the system reliable, fault-tolerant in the adversarial environment of MANET.

Overview: FTDN builds a logical tree-based structured network(Section 4.1), analyzes network dynamics (Section 4.2), replicates index information in the address publication process (Section 4.3), differentiates between occasional node failure (finds alternate paths avoiding the failure) and detects partition event (Section 4.4), and performs merging detection and merging (Section 4.5).

### 4.1. Logical Network Construction/LIDs Assignment and Key-Invariants

**Figure 2:**
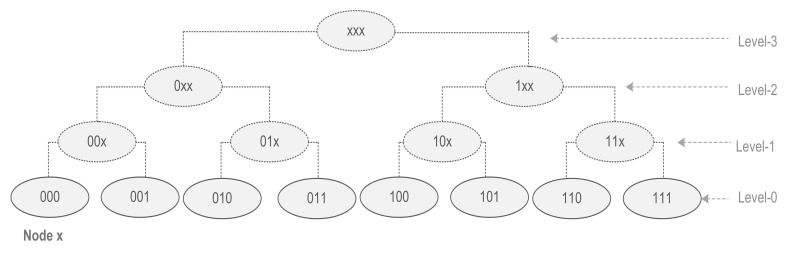
The Address tree.

For logical network construction (i.e., LIDs assignments), we are using existing algorithms in [14]. In network bootstrapping, joining nodes compute L-bit LIDs and build a virtual rooted binary tree, T(r), r represents the root of the tree, a node with the most miniature LID in the network is chosen as a root node in the tree. The L-bit longer LID forms a complete binary address tree with L + 1 levels. All the leaves remain at the same level in the address tree, and each inner vertex carries zero or two children. The leaves nodes represent the actual node (i.e., complete address), and the inner nodes, at a certain level, represent sub-trees (i.e., set of addresses with common prefix), as depicted in Figure 2. In general, an inner vertex in the address tree at a level-k represents a level-k sub-tree. The level-k sub-tree represents a set of network addresses/leaves (i.e., nodes) sharing a common prefix of (L-k) bits. A node can have at most L subtrees. Each node in the network belongs to one of these sub-trees(siblings). Similarly, all nodes that belong to a sub-tree share a common prefix.

For example, a tree T(x), rooted at x, is shown in Figure 2, node x has different sub-trees at each level, for example, at level-0 the sub-tree is S${}_{0}$(x) = {000}, i.e., contains a single leaf node (node x by itself), level-1 sub-tree is S${}_{1}$(x) = {001}, level-2 is S${}_{2}$(x) = {010, 011} and level-3 sub-tree S${}_{3}$(x) = {100, 101, 110, 111}. Each sub-tree at a certain level share a common prefix, i.e., (L- k) bits, for example, nodes in S${}_{1}$(x), S${}_{2}$(x) and S${}_{3}$(x) have common prefixes 00x, 0xx and 1xx, respectively. Furthermore, in the binary address tree a level-k sub-tree can have at most two level-(k-1) sub-trees.

The logical network construction algorithm (i.e., the address tree) preserves physical proximity and maintains a key invariant property called prefix sub-graph constraint.

Key-invariant: Adjacent nodes in the logical network (i.e., address tree) share a common prefix and represent a connected sub-graph in the physical topology. In other words, non-empty adjacent (i.e., with common prefix) sub-trees must be connected in the physical topology.

Formally, for a connected network, G = (V, E) with the address tree, T(x), rooted at x; there must be nodes, say, (i,j ∈ V) where i∈S${}_{k}$(x) and j∈S${}_{k-1}$(x) such that (i,j)∈E, where

{ ∃ i and j where i∈ S_k_(x) ∧ j∈ S_k−1_(x) | (i, j) ∈ E }

If the prefix subgraph constraint violates, disjoint instances of the address tree (i.e., network partition) would exist. FTDN exploits the key invariant and physical topological information (i.e., critical/non-critical nodes) and makes arrangements to detect the partition event (i.e., prefix subgraph constraint violation event) and differentiate between occasional node failure and partition. The key-invariant and the topological information play a vital role in finding alternate routing paths avoiding the failing nodes (i.e., occasional nodes failure without causing network partition), see Section 4.4 for detail.

Routing: FTDN exploits logical distances in the logical network and performs the forwarding decisions. For this purpose, a distance, δ, function based on Longest Common Prefix (LCP) match is used. The distance function δ(x,y) = L-LCP(x,y) computes logical distance (in terms of bits) and identifies the most significant bit that differs between the current node and the destination. Then, the next hop is determined by looking up the routing entry at the identified index (sub-tree) and packets are sent there, where L represents a total number of address bits used in the LIDs assignment. In other words, the distance function returns the sub-tree to which the destination address belongs and the node then looks up the next-hop table to find the next-hop towards the destination. In brevity, we omit diagrammatic and algorithmic details for the logical network construction/routing. For complete detail please see [14]. Our main objective is fault tolerance. The greedy forwarding and the prefix sub-graph constraint ensure the successful delivery of packets in the logical network.

### 4.2. Network Dynamics

**Figure 3:**
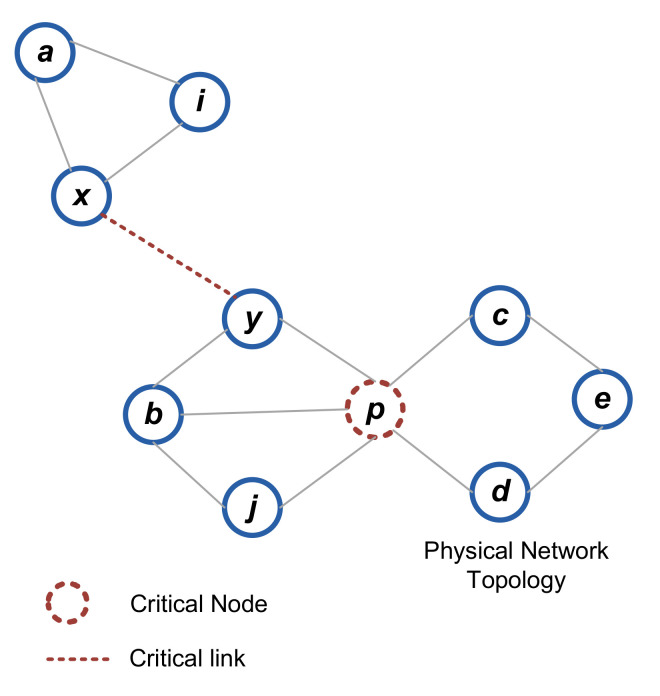
k-hop critical node/link scenario.

FTDN measures physical network dynamics (connectivity/dis-connectivity) using the concept of critical node(s)/link(s) [25,26] and adaptively makes arrangements (i.e., replicating data, partition detection, finding alternate paths avoiding the failed node(s), and reliable forwarding) in the logical networks to tolerate the faults. A node/link is said to be critical if its failure causes network partition. FTDN identifies critical nodes/links in a distributed manner by using k-hop common neighboring nodes. For example, as shown in Figure 3, the link(x,y) is said to be k-hop critical if its failure leaves node x and node y with disjoint k-hop neighboring nodes. For k = 1, the link(x,y) is 1-hop critical because 1-hop neighbors of node x and node y are disjoint. For k = 2, the link(x,y) is 2-hop critical, as there exist no common neighbors in the 2-hop neighbors of node x and node y. In this manner, the link(x,y) is considered a globally critical link because for k ≥ 1 there exist no common neighbors at all. Likewise, node p in Figure 3 is globally critical because there exists no common neighbor for k ≥ 1. If we increase the value of k then still there exist no common neighbors. Moreover, a globally critical node/link is always locally critical. Similarly, the failure of globally critical nodes triggers a partition event.

FTDN employs a k-hop localized distributed algorithm and computes critical nodes/links using k-hop topological information. For this purpose, nodes need to exchange the list of their k-1 hop neighbors’ information. In the simplest implementation (i.e., relying on the existing hello messages), nodes in the network periodically exchange the list of 1-hop physically connected neighbor nodes besides other information. In this manner, nodes attain 2-hop topological information. Formally, for a network G = (V, E), the neighborhood of a vertex v∈V is the induced subgraph of G consisting of all vertices adjacent to v and all edges connecting two such vertices. Similarly, the open neighborhood, N(v), of the vertex v consists the set of vertices adjacent to v excluding v, i.e., N(v) = {w∈N(v) : vw∈ E} and the closed neighborhood of v is N[v] = N(v)∪{v}.

Algorithm 1 computes critical nodes/links based on the localized knowledge available at nodes despite network-wide control information dissemination. For this purpose, (line: 1): nodes periodically exchange their open neighborhood in hello message. For any two adjacent nodes u and v across the link (u,v), node u and node v periodically exchange N(u) and N(v), respectively. (lines: 4–7): Node u and node v independently compare the exchanged lists, and if there exist common node(s), i.e., ( N[u]∩ N[v]∖ (u, v))≠{}, then node u and node v declare their status as non-critical (lines: 8–11): otherwise critical. The link between two critical nodes is treated as critical.
**Algorithm 1:** Critical node/link detection
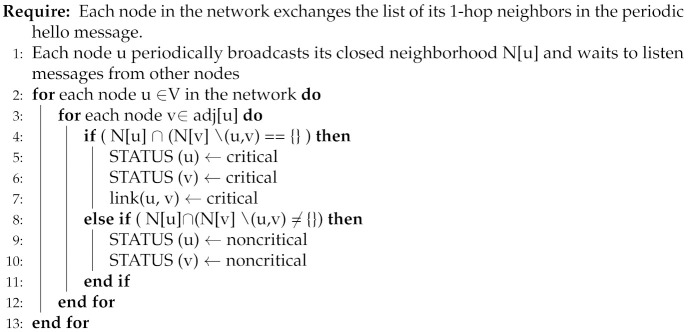


### 4.3. Address Publication

The address publication and lookup query resolution play vital roles in DHT networks and define overall network performance. Similarly, the resolution of lookup queries with minimum possible delay is imperative for successful communication. However, network dynamics such as AN failure, network partition prohibit resolution of lookup queries and exacerbate the delay. We believe that effectively replicating index information during the address publication at appropriate locations (i.e., based on network dynamics) would guarantee successful resolution of lookup requests; further, it would help to reduce lookup delays. However, replication should be dynamic, distributed, and scalable with minimum possible cost.

For this purpose, FTDN replicates index information in the address publication process across the critical regions (identified by Algorithm 1). FTDN performs replication and forwarding decisions at each node independently along the path to the intended AN using k-hop topological information (connectivity/dis-connectivity). Particularly, consider a newly joining node j; after computing LID node, j publishes and stores its index information (LID, UID pair) on AN. Node j determines its AN by applying a hash function over its UID, say v, generating a hashed value, say h(v), and stores index information on a node (i.e., A.N) with LID closest to h(v). For this purpose, node j broadcasts SMI (or SII) packets to be forwarded towards the intended AN. FTDN takes care of the SMI packets and replicates index information across all the critical links/nodes along the path to AN. If the actual AN fails/moves or the network gets partitioned, the index information remains accessible in the disjoint partitions, and nodes in the network could access node j’s index information from the replicas despite the actual AN. Algorithm 2 performs replication decisions using k-hop topological information. (line: 1): when an SMI/SII packet is received at node (i.e., k). (lines: 2–3): If node k finds itself closest to the hash valued (i.e., intended AN) then node k stores the mapping information and starts acting as designated AN. (lines: 4–6): otherwise, if there exists another node (i.e., m ∈ N(k)) closest to the intended AN and if the link (k,m) is critical, then node k keeps a copy of mapping information and forwards SMI to m. lines (7–9): alternatively, the SMI is forwarded to node m without making any replica.
**Algorithm 2:** Address Publication
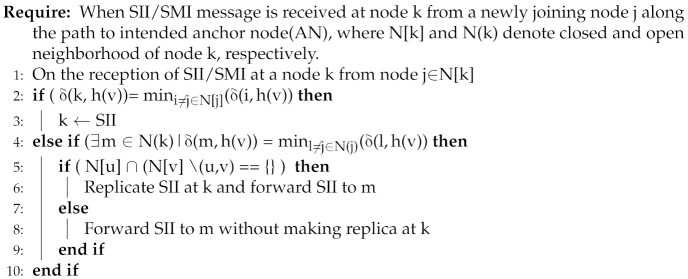


Impact on lookup: It is observed that the replication scheme significantly reduces lookup delays and improves lookup success ratio. The proposed address-publication targets critical regions (i.e., critical nodes/links) in the network, where these regions are more prone to failure (i.e., AN failure, partition, etc.); therefore, a trivial way is to replicate the index information across the critical regions so that index information would remain reachable despite actual AN fails or move/partition, etc. This ensures guaranteed access to index information in the adversarial environment and significantly improves the lookup success ratio with minimal delay. It is pertinent to mention that impact of the dynamic replication has been evaluated (using 3D and Chord structures) and found significant gains [25].

### 4.4. Partition Detection and Occasional Node Failure

A leaving/faulty node can cause two important events, i.e., partition detection and occasional node failure events. How to detect and differentiate these events need knowledge about network dynamics; we exploit network dynamics (critical/non-critical node/links) and the key-invariant property to effectively detect/differentiate these events.

CASE-I: Occasional node failure or node failure w.o.t causing partition (Finding alternate path avoiding the failed node)

FTDN uses a cross-design approach and finds alternate routing paths avoiding the failed node. Specifically, we consider logical network knowledge (i.e., key invariant property) and physical network connectivity information (i.e., critical/non-critical status). The cross-layer design approach finds and ensures a guaranteed available alternate path avoiding the failed node. FTDN finds an alternate path without creating any extra overhead and relies on the existing periodic hello messages. The parameters (i.e., key invariant and critical/non-critical) are computed independently at each node without any network-wide control information dissemination.

Key idea: if the leaving/fail node is non-critical, then there exists an alternate route in the physical network to reach across the failure, i.e., bypass the faulty node. Particularly, if an adjacent node u of a node v fails/moves, this would result in two rooted sub-trees in the logical network, i.e., T(r) and T(u). Node u and its siblings share a common prefix by the key invariant property and form a connected sub-tree rooted at node u, i.e., T(u). FTDN investigates the failure further in the physical topology using k-hop topological information and checks node u status as critical/non-critical. In occasional node failure (contrast to partition detection event), node v will find node u as non-critical; therefore, there exist common neighbors (i.e., in other words, there exist alternate paths avoiding the failure to reach nodes in T(u)). Algorithm 3 (lines: 3–5): finds such common neighbors and selects an appropriate next hop, k∈T(u). If there exist more than one common neighbor, the neighbor closest to the intended destination is chosen based on the logical distance in the logical network, i.e., k ∈ ( N(u) ∩ N(v)|(u,k) ∈ E ∧δ(k, d) = $min_{j\in N(u)\cap N(v)}\left(\delta \left(j,d\right)\right))$.
**Algorithm 3:** failure_handler(u,v,d) [Finding alternate path avoiding the failure]
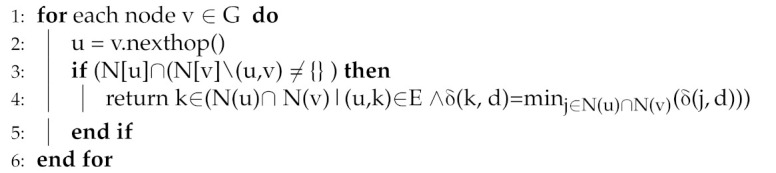


**Figure 4:**
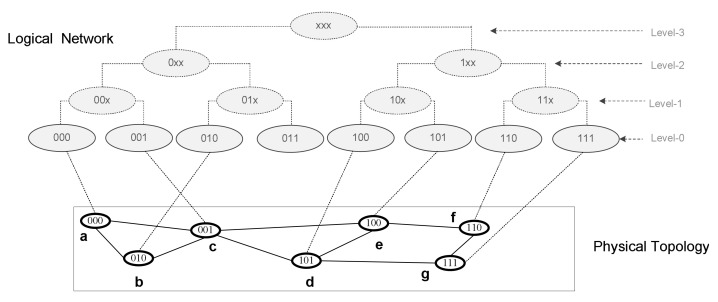
Physical vs. logical network.

The key invariant property and network dynamicity measurement (i.e., critical/non-critical) confirm the guaranteed availability of node k. FTDN finds alternate paths and bypasses the failing node in a fully distributed manner. As shown in Figure 4, nodes compute LIDs and form a logical tree over the Adhoc physical topology using the joining algorithm [14]. For instance, if the link(c,d) fails, node c finds node d as non-critical (since there exists a common neighbor, i.e., node e) and by the key-invariant node e and node d share a common prefix (i.e., 10x) thus form a connected subtree. In this manner, the proposed algorithm returns node e as the next hop to reach the subtree across the failure.

CASE-II: Partition Detection

If two adjacent critical nodes u and v in the network (i.e., logical tree T(r) rooted at a node r with the lowest LID in the network) do not hear each other for a specific time interval (i.e., Partition_Timer), a Partition_Event is triggered. In this case, the leaving neighboring node must be k-hop critical (i.e., k-hop neighbors of the leaving node u remains unreachable to node v and its neighbors N(v) if node u moves/fails) and causes network partition, i.e., there exist two distinct instances of the logical tree. The leaving node u and its siblings share a common prefix and form a connected sub-tree rooted at u, i.e., T(u) by key invariant property. Node v remains in the tree rooted T(r), where nodes in T(u) invoke the root election routine and select an appropriate root node, i.e., with the lowest LID. Thus, two valid instances of the logical tree (LS) with different roots exist. Algorithm 4 timely detect the partition event and ensures the existence of valid disjoint sub-trees. (line: 1–3): if two critical nodes with similar roots do not hear each other and the partition timer expires the partition event triggers. (lines: 4–5: nodes recover and reuse the LS by changing the roots).
**Algorithm 4:** Partition Detection
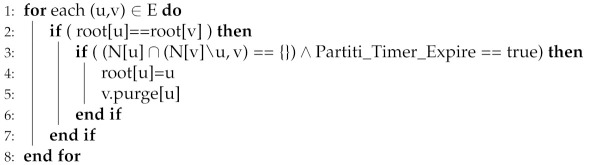


### 4.5. Merging Detection and Merging

As discussed in Section 2, merging physical topologies causes physically connected but logically independent networks; routing is performed on the logical structure (i.e., tree). Therefore, extra effort is needed for merging the disjoint logical structures (i.e., trees); merging logical networks should produce an evenly distributed LS (a connected logical network, i.e., a single-rooted tree ). Therefore, the merging process should consider measures such as LIDs reconfiguration and new root election, etc. Moreover, a smooth network working needs an evenly distributed LS (i.e., single root with the most miniature LID where all other (n-1) nodes LIDs form the sibling trees), how to merge two disjoint LSs (i.e., sub-trees) into an evenly distributed LS (i.e., singly connected rooted tree) with minimum cost is a challenging issue.

FTDN maintains root and rank information on every node in the network. FTDN exploits the existing periodically exchanging hello messages; the hello message contains root LID and rank value beside other information. If nodes across the network find different root LIDs in the periodic hello messages, nodes trigger merging detection events, and the actual merging process is initiated. Timely detection of merging detection events is needed to start a smooth merging process. The rank metric makes the merging process more efficient and cost-effective. Rank is an upper bound on the size of the rooted binary tree, and nodes compute rank value independently. Rank = (2${}^{h+1}-$1), h = L (height of the tree or address length, i.e., number of occupied address bits, excluding unused bits) is tree height. Similarly, the root node LID in a tree acts as representative/identity of a network, i.e., tree.

Merging: The merging process should produce an evenly distributed single-rooted tree with minimal cost. FTDN merges the smaller tree with the more extended tree. In other words, nodes belong to the smaller tree re-compute LIDs and correspondingly update root and rank information. Merging a smaller tree with the longer one makes the process cost-effective (i.e., would cause minimum possible reconfiguration cost). For this purpose, FTDN exploits the rank information and performs rank-based merging causing at most log(n) reconfiguration operations, i.e., re-computing LIDs, updating root and rank information. Algorithm 5 performs merging detection and merging process in a distributed manner. The hello messages carrying root, rank, etc., information are periodically exchanged by the nodes in the network. (Lines: 2): If a node u discovers a node v with a different root LID, node u perceives a merging detection event. Furthermore, (Lines: 3–8): a rank-based merging process is initiated where nodes in the smaller tree change LIDs, root, and rank information.
**Algorithm 5:** Merging
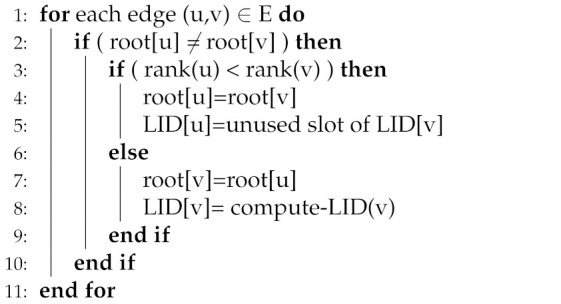


Rank-based merging significantly reduces reconfiguration costs. Since the ranks make sure to merge the smaller tree with the more enormous tree, smooth merging causes at most log(n) operations (LIDs to be updated) for n number of nodes, particularly, consider a node x in the network, each time node x’s LID was updated by the algorithm x must be in the smaller tree (due to rank metric). Therefore, the first time update in x’s LID would cause a tree to have at least two nodes. Similarly, a subsequent update (i.e., LID are only updated in the merging process) in the x’s LID at least double the size of nodes in the resulting tree, i.e., four nodes. In a tree implementation, at most log(n), total union operations are needed to merge n number of nodes. Therefore, most log(n) operations are needed to perform a smooth merging by the algorithm. In an arbitrary merging or blind merging (i.e., not ranked base), there can be at most (n − 1) merging events to unite n number of nodes, and at most O(n${}^{2}$) operation would be required for reconfiguration/LIDs updates. In other words, at most log(n) nodes would require reconfiguring LIDs.

### 4.6. Challenges

Synchronizing Replicas and Anchor Node: FTDN replicates and maintains mapping information across critical regions besides actual AN. This creates multiple replicas (i.e., ANs) alongside the actual AN. In this scenario, synchronizing the replicas and the actual AN is crucial. For this purpose, FTDN updates the mapping information maintained at the replicas as well as at the actual AN after a certain time interval, i.e., the Partition_Timer (the Partition_Timer is set to three times the hello interval). In this manner, a fresh copy of mapping information is available after every refresh period. However, the synchronization is achieved at the cost of extra transmission overhead.

Power constraints: FTDN identifies critical nodes/links and replicates the mapping information accordingly besides the actual AN. If the designated actual AN or the replicated node is a low-powered node then this would cause extra power consumption in the energy-constrained nodes. Therefore, measures are needed to cope with this issue.

Transmission Overhead: DHT routing protocols rely on periodically broadcasting hello messages and building logical structure network (i.e., nodes compute LIDs) over the ad hoc physical topology. Similarly, FTDN exploits the existing hello messages and exchanges control information such as 1-hop neighbors, root, and rank, among the neighboring nodes. This additional information in hello packets introduces extra transmission overhead. However, there is a trade-off between fault tolerance and overhead.

## 5. Simulation and Results Analysis

We implement FTDN in an open-source discrete event simulator, i.e., NS-2(2.34) [27] to evaluate the performance. Network parameters are tuned to standard values for both physical layer and link layer to simulate IEEE 802.11 along with a two-ray ground propagation model. The key objective is to create a contention-based MAC environment that best suits routing protocols in MANETs. Moreover, Constant Bit Rate (CBR) is used as a data traffic model over a user datagram protocol, and a random traffic model is used as a data pattern. Similarly, to avoid packet drops due to congestion in the multi-hop approach, the global traffic load is kept constant at 64 pkts/s. Likewise, mobility is another influential factor in the performance evaluation of routing protocols in MANET. Mobility compromises network connectivity (links) and is directly associated with network dynamics (nodes/links failures, network partition, merging, etc.). Mobility scenarios, based on random waypoint model, are created using BonnMotion2 [28], with nodes moving speed 0.5, 1.0, 1.5 and 2.0 (m/s) for different network sizes ( 50, 100, 150, 200). BonnMotion2 [28] ensures physical network partitioning and maintains network partitions, etc., records. Moreover, FTDN uses DART [14] routing mechanism in the implementation. The simulation parameters are listed in Table 2.

Performance comparisons have been made using following metrics with varying network size and speed.

- Lookup-Success Ratio (LSR): It is the ratio between total lookup queries (i.e., mapping request packets (MREQ) ) and the total lookup queries resolved successfully (i.e., total MREQ successfully entertained by receiving mapping reply (MREP)). LSR is measured using Equation (1).
  (1) $$\begin{array}{c} \mathrm{Lookup}\mathrm{Success}\mathrm{Ratio}(\mathrm{LSR})=\frac{\mathrm{Total}\mathrm{LookupRequests}\mathrm{Initiated}}{\mathrm{lookup}\mathrm{Requests}\mathrm{Successfully}\mathrm{entertained}} \end{array}$$
- End-to-End Lookup Delay: The average time taken by a lookup query to get resolved. In other words, the average time elapsed between MREQ and MREP encountered by a source node. It includes route discovery delay and queuing delay. It is measured in seconds. The E2E lookup delay is calculated using Equation (2).
  (2) $$\begin{array}{c} \mathrm{End}\mathrm{to}\mathrm{End}\;(E2E)\;\mathrm{Lookup}\mathrm{Delay}=\frac{\mathrm{Total}\mathrm{Lookup}\mathrm{Delay}}{\mathrm{lookup}\mathrm{Requests}\mathrm{Successfully}\mathrm{entertained}} \end{array}$$
- Normalized Overhead: It is the ratio between total number of transmission at the network layer and total number of lookup queries successfully resolved (i.e., total number of MREQ for which MREP is received).
  (3) $$\begin{array}{c} \mathrm{Normalized}\mathrm{Overhead}=\frac{\mathrm{No}.\;\mathrm{of}\mathrm{Transmissions}\mathrm{at}\mathrm{Network}\mathrm{Layer}}{\mathrm{Lookup}\mathrm{Requests}\mathrm{Successfully}\mathrm{entertained}} \end{array}$$
  Normalized overhead is an important metric to measure total routing overhead per successful lookup query. The normalized overhead per lookup request is measured using Equation (3).

### 5.1. Lookup Success Ratio

The lookup success ratio defines the overall fault tolerance capability of a DHT network. In the lookup process, a source node forwards MREQ (mapping info request) packets towards the AN and obtains mapping information (by receiving MREP (mapping reply) packet from the AN) to start a communication session with the destination node. Therefore, successful resolution of lookup requests is imperative to initiate actual data forwarding in DHT networks. However, network variations (node(s)/link(s) failures, network partition, MAC layer collisions, etc.) prohibit successful resolution of lookup. Similarly, the lookup resolution is directly associated with network size and mobility. The lookup success ratio decreases by increasing the network size. Because an increase in the network size increases the average hop count between a source and destination pair and the network traffic, this eventually causes packets collisions at the MAC layer (i.e., IEEE 802.11) and MREQ/MREP are likely to be delayed in a queue or dropped. Likewise, the lookup success ratio decreases as mobility increases because high nodes’ move speed compromises nodes connecting links (network connectivity) and introduces frequent network partition, merging, leaving/joining nodes, and failures (AN failures, etc.). These frequent and unpredictable changes introduce extra transmission overhead (due to the recurrent execution of joining, partition detection, and merging procedures), congestion at the MAC layer (losses of MREQ/MREP due to collision), and eventually, decrease lookup success ratio in DHT networks.

**Figure 5:**
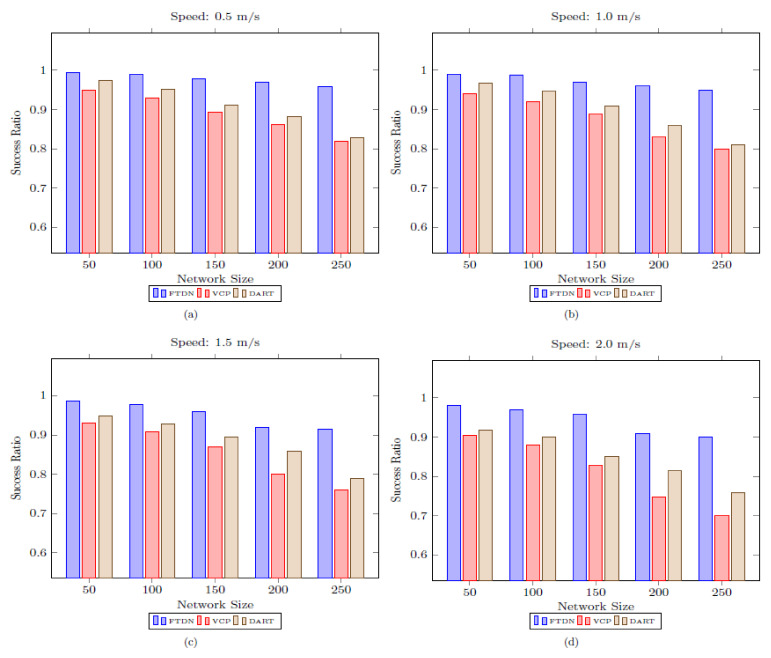
Lookup success ratio as a function of network size and speed.

Figure 5 shows lookup success ratio comparison against different network sizes and nodes moving speeds (the Figure 5a–d show the results at speed 0.5 m/s, 1.0 m/s, 1.5 m/s and 2 m/s respectively). In Figure 5, FTDN outperforms its counterparts and shows significant gains. Moreover, the impact of network size and mobility is lower in FTDN than VCP and DART; this shows the effectiveness of FTDN in highly mobile environments (i.e., in the presence of faulty nodes, partition, etc.) with an increasing number of nodes. In FTDN, the main factors in performance gain are dynamic replication (adaptively replicating mapping data across the critical regions in-network) and finding alternate paths to avoid failure. These measures ensure access to mapping information despite AN failure, routes breakages due to faulty nodes, and network partition. Moreover, these measures are fully distributed and rely on the local knowledge available to nodes. However, due to the recurrent execution of merging, LIDs reassignment, and increasing mobility and network size, a slight decrease in lookup success ratio is reported in Figure 5.

However, VCP and DART degrade lookup success ratio significantly as network size and mobility increase. Besides that, VCP maintains replicas (i.e., at predecessor/successor or physical 1-hop neighbors) and exploits a restricted ring search (restricted to 1-hop physical neighbors) to locate the replicas. However, due to mismatch problem [4,6] and network partitioning, the ring search not only fails, but introduces huge overhead (causes more packet collisions at MAC) and delay in the false attempts to locate replicas. Likewise, an increase in mobility and network size makes it worse and, eventually, the lookup success ratio drops. In DART, mapping data stored at the ANs are updated periodically; therefore, in case of failure or partition, the mapping data remains accessible after every refresh period, but if a lookup request reaches before the arrival of fresh copy, then the requests are buffered at the neighboring nodes (i.e., neighbors of the failed AN). This technique works well in small networks with lower mobility and provides access to mapping data despite network partition and actual AN failure. However, increasing mobility and network size limits the scope of such approaches because mobility increases the chances of failures (i.e., AN) and network partition (i.e., AN failures); this causes more lookup requests to be buffered and awaited to buffer overflow occurs, and requests are dropped.

**Figure 6:**
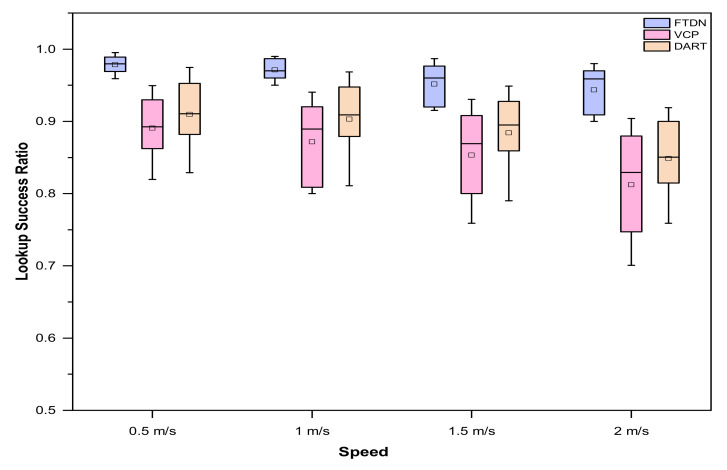
Lookup success ratio results, as boxplots, against different node moving speeds with varying network sizes.

Similarly, an increase in network size makes said phenomena worst, since the average hop count between a node and it is AN increase with increasing network size, so the periodic updates have to cover longer distances (i.e., updating mapping data); as a result, more and more requests are likely to buffer and be dropped. This tendency is apparent in Figure 5, where DART drops lookup success ratio as network size and mobility increases. However, as shown in Figure 5, DART performs well as compared to VCP. Figure 6 shows success ratio results against mobility as boxplots, indicating median and quartiles, where the small boxes show mean values. The boxplot analysis, along with comparison, shows consistent gains as compared to VCP and DART.

### 5.2. End-to-End Delay

Resolution of lookup queries with minimum possible E2E delay is imperative for successful routing in DHT networks and defines overall network performance, especially in the adversarial environment offered by MANETs. Similarly, network size and mobility are the influencing factors. Therefore, for a fair analysis, network size and mobility should be considered while analyzing E2E delay.

**Figure 7:**
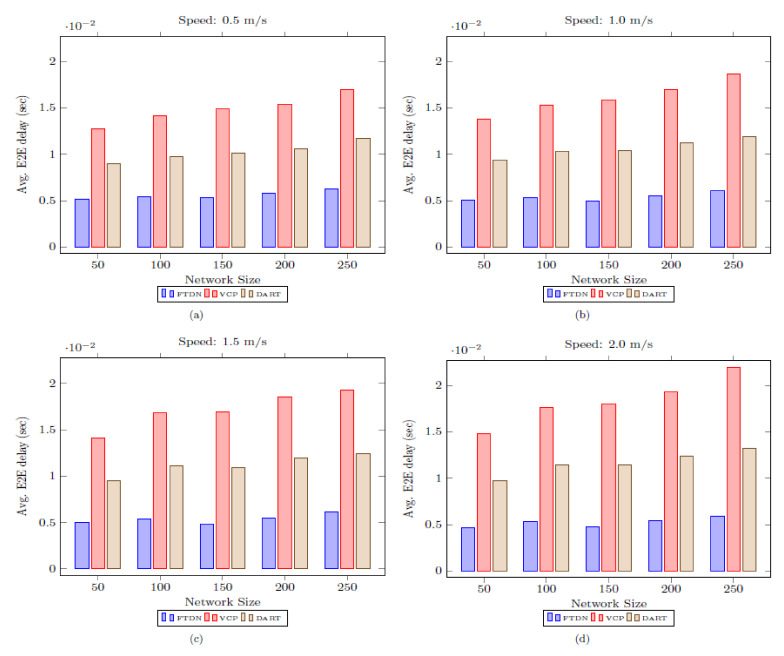
Average E2E lookup delay as a function of network size and speed.

Figure 7 shows E2E delay encountered by FTDN, DART, and VCP with varying network size and nodes moving speed (the Figure 7a–d show the results at speed 0.5 m/s, 1.0 m/s, 1.5 m/s and 2 m/s respectively). FTDN gains are significant as compared to DART and VCP. Since, in FTDN, the lookup queries are resolved by the nearby replicas despite being far from actual AN. Similarly, in case of actual AN failure or partition, the mapping information remains accessible comparatively at shorter distances than the original AN. Moreover, resolution of lookup requests by the nearby replicas instead of the designated AN reduces transmission overhead in FTDN. Eventually, this decreases contention at the MAC layer (i.e., in IEEE 202.11); these factors further reduce E2E lookup delay in FTDN compared to VCP and DART. Similarly, high mobility compromises network connectivity (links) and increases the probability of nodes/links failure and the existence of more critical nodes in the network. Therefore, FTDN deploys more replicas (i.e., since, in FTDN, replication decisions consider network dynamics and adaptively replicate mapping info across the critical regions) and, the lookup queries would get resolved by the nearby replicas, even in highly mobile environments. So, FTDN is a better choice for highly mobile environments. However, a slight increase in overhead and delay due to recurrent partition detection and merging schemes. However, the case is different in VCP and DART since VCP does not consider physical network dynamics in the replication process and offers no resistance to mobility. Specifically, in highly mobile environments, the restricted ring search used in VCP not only fails to discover replicas but introduces massive delay due to the false attempts by locating the replicas. Similarly, in DART, due to frequent failures (nodes/links, partition, etc.), more and more lookup queries are needed to be buffered; this causes a buffer overflow, and the forwarded lookup quarries get lost before reaching the new selected AN. This worsens with an increase in network size (i.e., an increase in network size also increases average shortest path length; therefore, SMI packets have to cover longer distances in periodic updates. Therefore, VCP and DART encounter considerable delays in the lookup process as mobility and network size increase. However, for small networks with low mobility, DART outperforms VCP.

**Figure 8:**
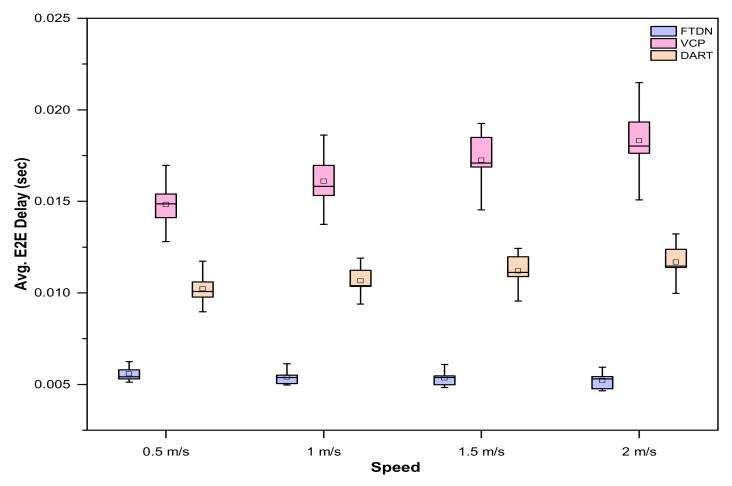
E2E delay results, as boxplots, against different node moving speeds with varying network sizes.

Figure 8 provides E2E delay comparison using boxplots against different nodes speed with varying network size. The data distribution further endorses FTDN’s significant gains.

### 5.3. Normalized Overhead

Normalized overhead defines network scalability and shows transmission overhead per lookup request successfully entertained. In general, the operations carried out by a protocol produce overhead, but factors such as network size, mobility, etc., are directly associated.

**Figure 9:**
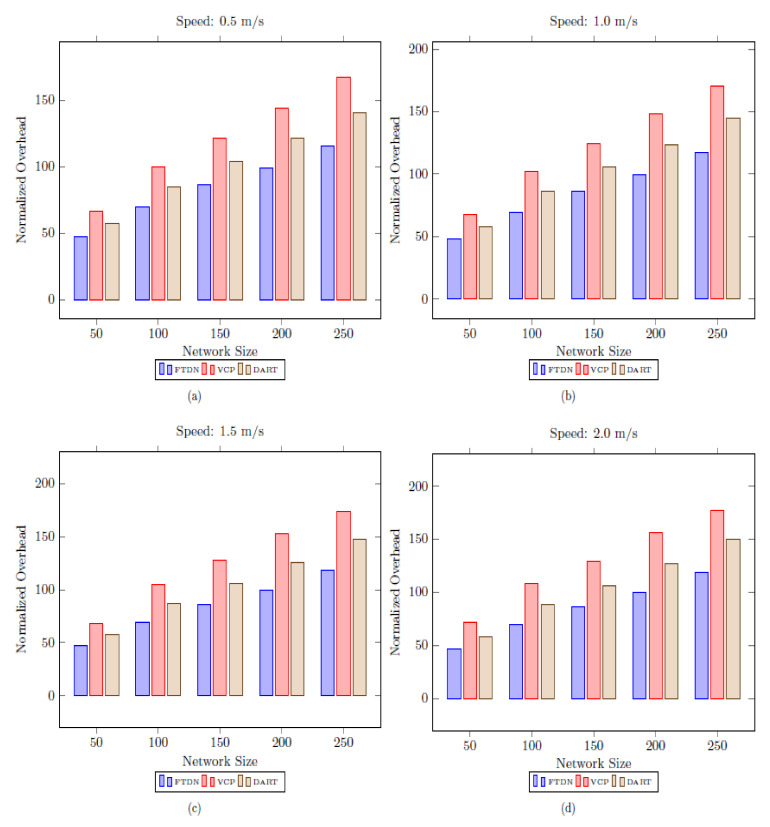
Normalized overhead as a function of network size and speed.

Therefore, we provide an overhead lookup comparison using varying network sizes and nodes’ moving speed. As shown in Figure 9, FTDN gains are significant as compared to VCP and DART (the Figure 9a–d show the results at speed 0.5 m/s, 1.0 m/s, 1.5 m/s and 2 m/s respectively). The dominant factors in overhead reduction are the dynamic replication of mapping information and alternate paths avoiding faulty nodes. These measures make possible resolutions of lookups with minimum possible overhead in the presence of faulty nodes (i.e., AN failures, network partition) since nodes can find mapping data at nearby replicas despite forwarding lookup requests too far away distant ANs. However, the case is different in VCP and DART; for example, deploying and locating replicas (i.e., ring search) in VCP need extra transmissions and cause more overhead. Similarly, in DART, lookup queries are resolved far away from actual AN (i.e., no replication); that is why DART produces more overhead than FTDN. Moreover, growing network size and increasing mobility produce more overhead in all protocols, but FTDN still performs well than VCP and DART. A slight increase in FTDN, with an increase in mobility, is due to the recurrent execution of LS recovery, LIDs reassignment, and merging processes; similarly, growing network size increases an average number of hope counts to reach AN; eventually, this increases overhead per lookup request. However, this impact is lower in FTDN as compared to VCP and DART as depicted in Figure 9.

**Figure 10:**
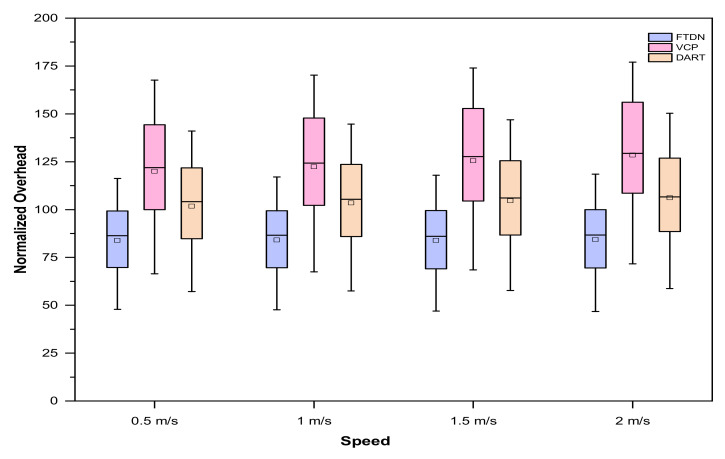
Normalized overhead results, as boxplots, against different node moving speeds with varying network sizes.

Boxplot analysis comparison, with varying network sizes and speed, shown in Figure 10, shows consistent gains in FTDN as compared to VCP and DART.

## 6. Conclusions

In this paper, we outline the challenges to address the problem of fault tolerance in DHT networks and provide a suite of distributed mechanisms to solve the identified issues. We have described that network dynamics (i.e., node(s)/link(s) failure, network partition, merging, etc.) halt communication in the DHT network and limit the scope of such protocols in the adversarial environment offered by MANET.

In particular, a fault-tolerant DHT-based (FTDN) routing protocol is proposed for MANET. FTDN builds and maintains a logical tree-structured network over MANET’s ad hoc physical topology and eliminates flooding at the control and data plans. FTDN, using a cross-layer design approach, exploits *k*-hop topological information (i.e., physical network connectivity/dis-connectivity) along with the philosophies of detection, recovery, avoidance, and redundancy. Moreover, FTDN considers pre-failure measures (i.e., measuring network dynamics, replication, and partition detection) and post-failure measures (i.e., finding alternate paths avoiding the failure, merging detection, merging, i.e., reconfiguration). These measures ensure DHT-network availability and make the system reliable, fault-tolerant in adversarial environments. Performance evaluation confirms the effectiveness of the solutions provided.

In the future, we would like to extend the performance analysis in two directions: (1) current implementation considers 1-hop topological information; in the future, we would like to use a different variation of *k*. (2) To implement the proposed solutions in other logical structures by considering different performance parameters (throughput, etc.). 

## Figures and Tables

**Table 1:** Definitions/Abbreviations used in DHT routing.

Term/Symbol	Description
Anchor Node (AN)	A node that holds the mapping information of other nodes. A source node has to retrieve the mapping info. from the AN before initiating a data session with destination. Any node in the network can act as AN.
Logical Identifier(LID)	A unique ID that identifies a node in the logical structured network and, represents the node relative position in the logical structure.
Logical Space (LS)	The LIDs are drawn from the LS. The LS represents a range of valid LIDs and forms a structured logical network i.e., tree, chord etc.
Logical Space Portion (LSP)	Nodes in the logical network maintain a disjoint portion of LS called LSP. The LSP denotes relative position of a node in the logical network and acts as LID.
Mapping Information/Index Information	The mapping info. (i.e. (UID, LID) pair) provides mapping between permanent identifier (IP/MAC) and LID. The mapping information are maintained at AN.
Logical Network (LN)	The interconnection of nodes based on their LIDs is called logical network.
Store Mapping Info. (SMI/SII) packet	After computing LID, a joining node stores its mapping information on AN by forwarding SMI packets in the network.
Universal Identifier (UID)	A unique identifier of a node that remains constant in the network life time. UID can be the IP or MAC address.
Mapping Request (MREQ) packet	A source node retrieves the mapping info. from the AN by sending MREQ packets in the network.
Mapping Reply packet (MREP)	The AN shares the requested mapping info. by sending MREP packet.
N(v)	It represents 1-hop neighbors of node v excluding node v, called open neighborhood of node v.
N[v]	It shows 1-hop neighbors of node v including node v, called close neighborhood of node v.
LSR	It stands for Lookup Success Ratio.
E2E	E2E stands for End-to-End lookup delay.

**Table 2:** Simulation Parameters.

Parameters	Values
No. of Nodes	[50, 100, 150, 200]
Radio range	100 m
Network area	1000 m × 1000 m
Data rate	64 pps
Simulation time	500 s
Speed	[0.5, 1.0, 1.5, 2 (m/s)]
Topology Connectivity/Generation	BonnMotion2
Propagation Model	Tow-Ray Ground
Avg. no. of network partitioning	5 times
No. of flows	12
Traffic Model	Random Traffic pattern
Routing Protocols	DART
Mobility Model	Random way point
